# Reactive Deep Eutectic Solvent for an Eco-Friendly Synthesis of Cellulose Carbamate

**DOI:** 10.3390/polym16060757

**Published:** 2024-03-09

**Authors:** Vincenzo Algieri, Loredana Maiuolo, Debora Procopio, Paola Costanzo, Fiore Pasquale Nicoletta, Sonia Trombino, Maria Luisa Di Gioia, Antonio De Nino

**Affiliations:** 1 Department of Chemistry and Chemical Technologies, University of Calabria, Via P. Bucci, Cubo 12C, 87036 Rende, CS, Italy; vincenzo.algieri@unical.it (V.A.); maiuolo@unical.it (L.M.); paola.costanzo@unical.it (P.C.); 2 Department of Pharmacy, Health and Nutritional Sciences, University of Calabria, ed. polifunzionale, 87036 Rende, CS, Italy; debora.procopio@unical.it (D.P.); fiore.nicoletta@unical.it (F.P.N.); sonia.trombino@unical.it (S.T.)

**Keywords:** cellulose, carbamate, derivatization, reactive deep eutectic solvents, erbium trichloride, green chemistry

## Abstract

The limited solubility of natural cellulose in water and common organic solvents hinders its diverse applications, despite being one of the most abundant and easily accessible biopolymers on Earth. Chemical derivatization, such as cellulose carbamate (CC), offers a pathway to enhance both solubility and industrial processability. In this study, CC was synthesized by exploiting a novel type IV deep eutectic solvent (DES) composed of erbium trichloride and urea. This DES was shown to be not only an environmentally friendly reaction medium/catalyst but also actively participated in the synthetic process as a reagent. The resultant cellulose carbamate samples were characterized through FT-IR and elemental analysis. A nitrogen content value of 1.59% was afforded determining a degree of substitution corresponding to a value of 0.19. One of the key scientific advancements lies in the preparation of cellulose carbamate using a straightforward and cost-effective method. This approach utilizes non-toxic compounds, aligning with the principles of green chemistry and contributing to sustainable development in cellulose derivative production.

## 1. Introduction

**Figure 1:**
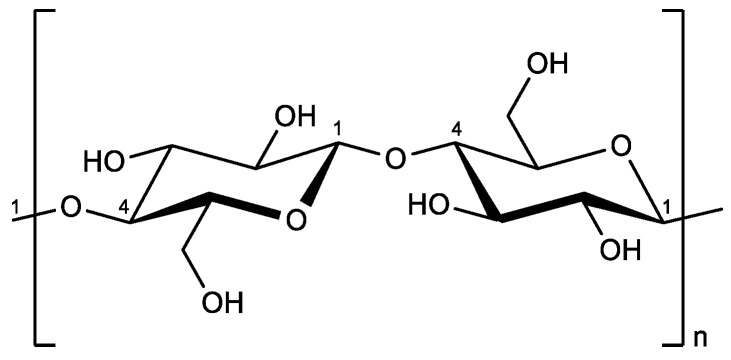
Chemical structure of Cellulose.

The accumulation of synthetic polymers in the environment has led to significant environmental pollution issues, which can only be mitigated by exploring biodegradable and non-toxic biopolymers [1]. In this context, industries play a crucial role in focusing on sustainable resources and increasing the use of renewable raw materials. Natural cellulose (Figure 1), due to its abundance and versatility as a biopolymer, represents a significant resource for various applications [2,3]. Its easy accessibility, biocompatibility, high mechanical stability, and unique physiochemical properties make it suitable for applications in biomedical science, environmental science, and sustainable packaging [4,5]. Despite the enormous potential of cellulose, only a small fraction is currently utilized for further processing [6]. This is attributed to its complex inter- and intramolecular hydrogen bonding network, leading to reduced solubility in water and most common solvents [7,8,9]. The OH groups at position 6 are the ones responsible for intermolecular bonding in cellulose. Consequently, the accessibility of these groups is the limiting factor for cellulose solubility.

Numerous solvents have been explored and/or have been introduced into industry to improve cellulose solubility, such as *N*,*N*-dimethylacetamide/lithium chloride (DMAc)/LiCl, dimethyl sulfoxide/tetrabutylammonium fluoride (DMSO/TBAF), *N*,*N*-dimethylformamide/dinitrogen tetroxide (DMF/N_2_O_4_), *N*-methyl-morpholine-*N*-oxide (NMMO), and ionic liquids [10,11,12,13,14,15,16,17]. However, these methods often present limitations, especially in terms of cost and environmental impact, necessitating the development of more cost-effective and environmentally friendly alternatives.

**Scheme 1:**
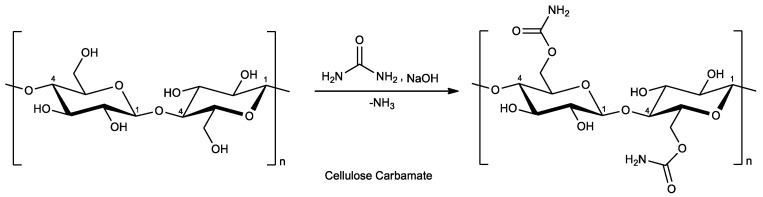
Chemical modification of cellulose into cellulose carbamate (CC).

A frequently used approach to enhance cellulose solubility is pre-chemical modification, such as converting it into cellulose carbamate (CC), as shown in Scheme 1. This modification renders cellulose biodegradable and biocompatible, making it an eco-friendly material with applications in absorbent products, food packaging, chromatography, and fireproof products [18,19,20,21,22,23,24] The conventional “CarbaCell” process involves heating cellulose with urea, resulting in cellulose carbamates with a nitrogen content (N%) of around 1–2.5% (Scheme 1) [25]. However, this process faces challenges in industrial reproducibility due to rigorous conditions, including high temperatures, long reaction times, and the use of a catalyst [26].

In response to the need for innovative and efficient methods for cellulose carbamate preparation, it is worth exploring environmentally sustainable solvents and methods [27,28,29,30,31,32]. Ionic liquids (ILs), which are traditionally considered environmentally friendly and very versatile non-conventional solvents for chemical transformations [33,34,35,36,37], have limitations in terms of biodegradability and sustainability [38,39,40,41]. Recently, Deep Eutectic Solvents (DESs) have gained attention as potential green solvents for biomass processing, offering biocompatibility and applications in bio-pharma industries [42,43,44,45,46,47,48,49,50,51,52,53]. Several studies have emerged in which DESs are used as solvents for the derivatization of cellulose and to produce various types of nanocelluloses [54,55,56,57]. The IV-type class of Deep Eutectic Solvents formed using hydrated metal salts as a cationic component have been widely used in the treatment of biomass [36,51]. Recently, a DES based on dimethylurea and zinc chloride was proposed for the preparation of cellulose methyl carbamate [58]. Nevertheless, zinc chloride is known to be a toxic salt, capable of accumulating if improperly disposed of [59,60,61,62,63,64].

Encouraged to seek out more eco-friendly solutions and to address concerns about toxic salts, we propose the possible combination of erbium trichloride (ErCl_3_) and urea in different molar ratios to form a type IV DES. In this regard, erbium salts have emerged as a sustainable catalytic solution for a series of organic transformations that require the presence of Lewis acid catalysts [65,66,67]. Erbium trichloride (ErCl_3_) was chosen for its cost-effectiveness, low toxicity, and versatility [65,68,69,70]. This Lewis acid was combined with urea, which is not only one of the most used components in DESs but also represents a promising chemical for cellulose modification as it is a low-cost, readily available, and non-toxic compound [71]. The developed reactive deep eutectic solvent (RDES), acting as a reaction medium, reagent, and catalyst, holds promise in improving the environmental sustainability of cellulose carbamate production.

## 2. Materials and Methods

### 2.1. Materials

Microcrystalline cellulose, Avicel PH-101 (MCC, Mw = 53,470, Mn = 24,235, DP = 350) was purchased from Sigma–Aldrich (St. Louis, MO, USA). Urea (99.7%) and Er (III) chloride hexahydrate (ErCl_3_·6H_2_O, 99.9%) were purchased from Merck and used as received without further purification. Deionized water was used in all experiments. The heating necessary for the preparation of DESs was provided using a Rotavapor Heidolph Laborota 4000 (Heidolph Instruments Italia, Milano, Italy). Drying was carried out using a Vismara Vacuum Oven 65 stove. The product, after recovery by washing with a solution of HCl 12 M and filtration, was dried using an Edwards XDS5 diaphragm pump (Crawley, West Sussex, UK). Ultrasonic irradiation was carried out using a Hielscher VP 2005 Sonicator (Hielscher Ultrasonics GmbH, Teltow, Germany)

### 2.2. Preparation of Reactive Deep Eutectic Solvents (RDESs)

#### 2.2.1. Preparation of DES-1 Using the Vacuum Evaporation (VE) Method

ErCl_3_ (0.5078 g, 0.00185 mol) and urea (0.3719 g, 0.0062 mol) in a 3:10 molar ratio were weighted in separate vials and dissolved in water. Then the two solutions were mixed together in a round-bottom flask and dried using a rotary evaporator (at 50 °C) until a clear, homogeneous pink liquid was formed (Table 1). The DES was stored in a desiccator.

#### 2.2.2. Preparation of DES-2 Using the Ultrasound-Assisted (US) Method

The two components, ErCl_3_·6H_2_O and urea, were mixed in a 10 mL glass flask in the molar ratios indicated in Table 1. The glass flasks were placed in a KQ-300E ultra-sonic bath (Kunshan ultrasonic instruments Co., Ltd., Kunshan, China) with an ultra-sonic input power of 300 W and a frequency of 40 kHz. The mixture formed a clear and homogeneous pink liquid at room temperature after up to 1 h of ultrasonication. The final temperatures of the ultrasonic bath could reach up to 50 °C due to the long duration of ultrasonication. DES-2 was stored in a desiccator.

#### 2.2.3. Preparation of DES-3 Using the Heating and Stirring (HS) Method

ErCl_3_·6H_2_O (0.5074 g, 0.00185 mol) and urea (0.3743 g, 0.00623 mol) in a 3:10 molar ratio were placed in the same vial and water (17 µL, 0.95 µmol) was added. The vial was placed in a water bath at 60 °C and stirred magnetically until the formation of a clear pink liquid was observed (see Table 1). The DES was stored in a desiccator.

#### 2.2.4. Preparation of DES-4 Using Both Ultrasonication and Vacuum Evaporation Methods

DES-4 was prepared by placing 1.01 g of ErCl_3_·6H_2_O and 0.74 g of urea (molar ratio 3:10) in a round-bottom flask and combining both ultrasonication-assisted and vacuum evaporation methods. The reaction was first assisted by ultrasound for 1 h and then it was stirred using a rotary evaporator at a temperature of 40 °C for 1 h. The pink liquid (Table 1) was finally preserved in a desiccator.

All the DES samples were kept at room temperature for an extra 24 h after their preparation to ensure the formation of the liquid. All the prepared DESs were characterized by Differential Scanning Calorimetry (DSC) analysis, Fourier Transform Infrared Spectroscopy (FTIR), and Polarized Optical Microscopy (POM).

### 2.3. Differential Scanning Calorimetry (DSC) Analysis of RDESs

DES-1–DES-4 were characterized by Differential Scanning Analysis (DSC) performed using a DSC instrument (DSC 200 PC Netzsch, Wittelsbacherstraße 42, Selb, Germany). The temperature ranged from −80 °C to 350 °C, at 10 °C/min, after equilibration for 5 min at −80 °C. The experiments were performed under a nitrogen atmosphere (50 mL/min), with 15 mg of the sample in aluminum pans with covering lids.

### 2.4. Polarized Optical Microscopy (POM) Analysis

A small droplet of the prepared DESs was deposited on a microscopic slide for observation at a magnification of 10×. The Nikon ECLIPSE LV100N polarizing microscope (Nikon Corporation, Shinagawa Intercity Tower C, Tokyo, Japan) coupled with the Nikon DS-Fi2 camera was used for recording the polarized light image. The absence of a solid crystalline structure is evidenced by a polarized light image that is totally black.

### 2.5. Fourier Transform Infrared Spectroscopy (FT-IR) Analysis

The prepared DESs were analyzed by means of the KBr sheet. Infrared (FTIR) spectra were recorded using an FTIR Perkin-Elmer 1720 spectrophotometer over the 4.000–400 cm^−1^ range at a rate of 0.5 cm/s. Fifty scans were recorded, averaged for each spectrum, and corrected against ambient air as the background.

### 2.6. Synthesis of Cellulose Carbamate (CC)

#### 2.6.1. Experiment 1: Reaction in DES-4 Using VE

DES-4 was prepared following the procedure reported above. Subsequently, 0.21 g of microcrystalline cellulose (a 10-fold molar excess of urea compared to the cellulose anhydroglucose unit) suspended in distilled water (2.23 mL) was introduced into the flask containing DES-4. A glass rod was employed to ensure the uniform mixing of cellulose in DES. The reaction mixture was subjected to continuous stirring using a rotary evaporator at a reduced pressure at 60 °C for 1 h, and then dried in an oil bath at 150 °C for an additional hour. The resulting product was dry and adhered to the flask walls.

After cooling, the recovery of the solid material was carried out by washing with 20 mL of a 1 M HCl solution, followed by filtration and subsequent washing with distilled water to remove the unreacted residual DES components. The product was filtered and dried using a vacuum pump, resulting in a white solid material.

IR νmax(KBr): amide N-H, 3300; amide C=O, 1716.34 cm^−1^.

#### 2.6.2. Experiment 2: Reaction in DES-4 Using VE and US

In a 10 mL round-bottom flask, DES-4 was prepared. Then microcrystalline cellulose (0.1030 g) and distilled water (2.23 g, 0.124 mol) were introduced. Gentle mixing was performed by manually shaking the flask. The reaction was assisted by ultrasound for 1 h. Subsequently, the mixture was stirred using a rotary evaporator under reduced pressure at 50 °C for 1 h. The product was dried in an oven at 100 °C for 24 h. After cooling to room temperature, the recovery and purification of the CC derivative were carried out as described in experiment 1. The recovered product was finally dried, resulting in a gray-colored powder.

#### 2.6.3. Experiment 3: Reaction in DES-4 under US in the Absence of Water

The synthesis was conducted in the absence of water, and therefore, microcrystalline cellulose (1.0946 g) was dried to eliminate traces of residual moisture until a constant weight was reached. In a test tube, previously dried microcrystalline cellulose (0.1015 g) was added to DES-4. The reaction was assisted by ultrasound for 1 h. The product was dried in an oven at 100 °C for 24 h. The test tube was allowed to cool to room temperature, and the final recovery and purification of the CC derivative was carried out as described in experiment 1. The obtained product was dried to afford a white-colored solid material.

#### 2.6.4. Experiment 4: Reaction in the Absence of DES-4 under US

To a 10 mL round-bottom flask dried containing cellulose (0.10 g) suspended in water (6.70 g, 0.372 mol), urea (0.317 g, 0.00062 mol) was added. The reaction mixture was subjected to continuous stirring using the rotary evaporator at a reduced pressure at 60 °C for 1 h, and then dried in an oil bath at 150 °C for an additional hour. The product was then dried in an oven at 100 °C for 24 h and finally allowed to cool to room temperature. The final recovery and purification of the CC derivative was carried out as described in procedure 1 affording a white-colored product.

### 2.7. FT-IR Analysis of CC

Cellulose carbamate (CC) was characterized by means of a Fourier Transform Infrared (FT-IR) spectrometer (Jasco 4200, Cremella, LC, Italy) and compared with native cellulose. FT-IR spectra were performed using a Jasco 4200 spectrometer using the KBr disk technique. Spectra were obtained in the 400–4000 cm^−1^ range.

### 2.8. Elemental Analysis of CC

Elemental analysis was carried out to determine the nitrogen content (N%) by a vario MICRO CHNS V4.0.10 analyzer from Elementar (Langensebold, DE, Germany), where the samples were burned within a jet injection of oxygen. The gaseous components were purified and separated on a TPD column before quantification with a thermal conductivity detector. Helium was used as a carrier gas.

Percentages of the elemental analysis for C, H, and N elements are reported in Table 2. The degree of substitution (DS) was calculated from the nitrogen content of CC via the following Equation:

$$DS=\frac{N\times 162.15}{14\times 100-\left(N\times 43\right)}$$

where *N* is the nitrogen content, 162.15 is the molecular weight of the anhydroglucose unit (AGU) of cellulose, 14 is the atomic weight of the nitrogen, atom and 43 is the molecular weight of the carbamate group [72].

## 3. Results

### 3.1. DES Formation

At the beginning of our investigation, we considered different procedures for preparing RDESs to ensure reproducibility: the vacuum evaporation method (VE), the heating and stirring method (HS), and the ultrasound-assisted method (US). These diverse approaches may yield distinct outcomes. In fact, variables like temperature, pressure, and water content, which are influenced by these methods, can shape the final composition of the DESs, thus affecting their physicochemical properties and stability [73]. Therefore, initially, we applied the vacuum evaporation method, where all available water was removed, leaving only the water interacting with the DES components in the system [74,75]. To achieve this, erbium trichloride and urea in different molar ratios were dissolved in water and stirred using a rotary evaporator under reduced pressure at 50 °C for 1 h until a clear, homogeneous pink viscous liquid was formed. ErCl_3_ in its anhydrous form was found unsuitable for the intended purpose. The stoichiometric ratios investigated in combination with urea (1:1 or 1:3) did not produce recognizable DES due to the presence of solid particles of the salt and rapid solidification of the mixture after cooling (Table 1, entries 1, 2). Similarly, the use of the hexahydrate form of erbium trichloride, with 1:1 and 1:3 molar ratios, resulted in the formation of pink and highly viscous liquids with particles in suspension, making them unsuitable for easy handling and reaction (Table 1, entry 3). Instead, a clear, pink, and homogeneous liquid was obtained when using erbium chloride hexahydrate and urea in a 3:10 molar ratio (Table 1, entry 4, DES-1). The resulting DES was then dried in a desiccator containing silica gel until a constant product weight was achieved.

In a further attempt, the DES was prepared using the ultrasonic bath method [76]. As widely reported in the literature, the use of ultrasound allows for obtaining a more homogeneous mixture in a shorter time due to the phenomenon of cavitation [77]. Nevertheless, the DES was obtained as a clear and homogeneous pink viscous liquid only after 60 min of ultrasonication of the components (Table 1, entry 6, DES-2).

**Figure 2:**
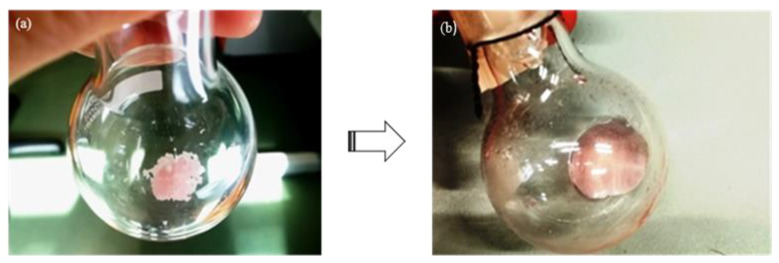
DES-4 prepared using both vacuum evaporation and ultrasound-assisted methods. (**a**) ErCl_3_·6H_2_O and urea, solid reagents in contact; (**b**) formation of the DES-4 after US and heating.

The preparation of the DES was also attempted using the heating and stirring method. In this case, 20% of water was added to a mixture of ErCl_3_·6H_2_O and urea (in a 3:10 molar ratio). Subsequently, the components were placed in a round-bottom flask and subjected to magnetic stirring in a 60 °C water bath for 1 h, resulting in the formation of a clear, viscous pink liquid (Table 1, entry 7, DES-3). A final experiment (Table 1, entry 8, DES-4) was conducted in the absence of water by exploiting the efficiency of both ultrasound and vacuum evaporating. The mixture, consisting of erbium trichloride and urea, underwent ultrasonication for 1 h and was then stirred under vacuum at reduced pressure at 60 °C for an additional 1 h. Even in this case, the product appeared as a pink, viscous liquid (Figure 2b).

Taking into consideration the results obtained from previous tests, we inferred that erbium hexahydrate combined with urea can form a Deep Eutectic Solvent (DES) when a molar ratio of 3:10 is employed. Additionally, it is observed that the presence of additional water for DES formation is not necessary, and both heating and ultrasound simplify the product formation.

### 3.2. DES Characterization

**Figure 3:**
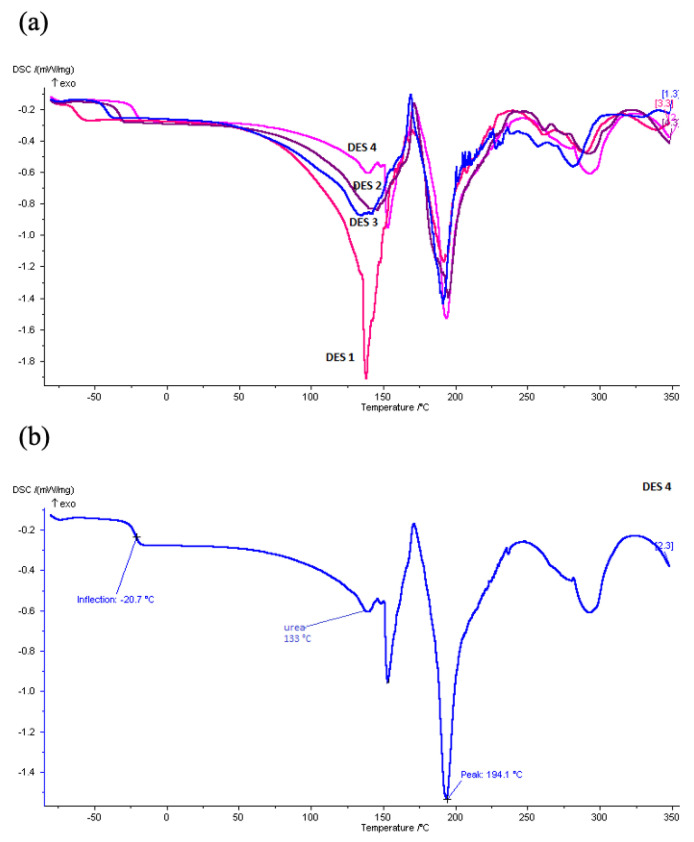
DSC thermograms of the (**a**) prepared DESs and (**b**) single DES-4.

The formation of the liquid mixture when the two components were combined in a 3:10 molar ratio was supported by observation and POM imaging. The POM image revealed that the DES was formed because a black image was visible, showing that the sample was completely amorphous, and no crystals remained in the system. The successful formation of the DESs was further demonstrated by measuring the melting point or glass transition temperature of the DESs by differential scanning calorimetry (DSC) analysis as shown graphically in Figure 3.

From the thermograms obtained, it was possible to denote how the melting point of the eutectic is different than its individual components, erbium trichloride (776 °C) and urea (133 °C). According to Abbott and collaborators, this change is dependent on the reticular energies of the salt and hydrogen-bond donors (HBD), the way in which the couple interacts, and the entropy changes deriving from the formation of a liquid phase [78,79]. Furthermore, by comparing the four thermograms and observing the intensity of the peaks due to urea as well as the eutectic point, we could deduce that the DES-4 was the best-prepared one. In fact, DES-4 showed a less intense peak relative to urea than that observed in the other thermograms (Figure 3b). This indicated a lower amount of urea in the free state, and consequently, its better coordination with erbium. In this context, the lack of DES formation when using anhydrous erbium trichloride underlines how crystalline water represents a fundamental feature for the formation of this category of DES. Therefore, water molecules are real ligands coordinated at the center of the erbium atom or associated with free chloride ions [80].

**Figure 4:**
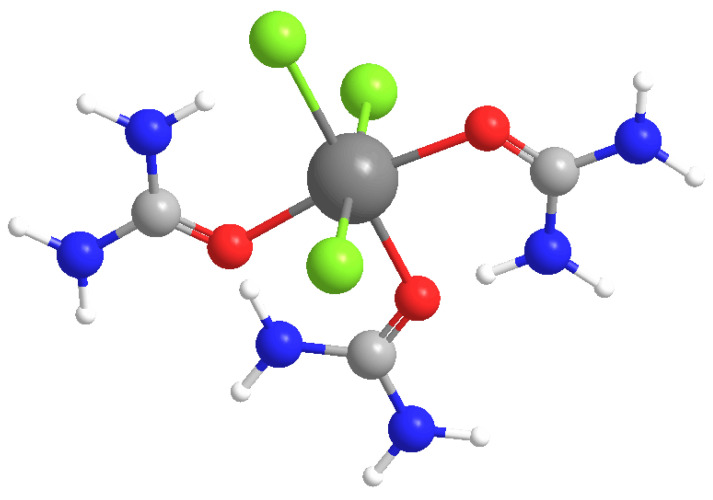
Hypothesis of the hexacoordinated structure of DES-4.

From the thermogram in Figure 3b, it is also possible to establish how DES-4 has a high glass-transition temperature (−20.7 °C) compared to the other experiments, thus confirming the stability of the solvent obtained. These observations led us to hypothesize a structure of the DES formed: erbium is hexacoordinate [81], that is, it forms natural binding interactions with the three chloride ions and coordinative interactions with three urea molecules, assuming a hypothetical structure that is represented in Figure 4.

FTIR characterization of the synthesized DES was conducted in order to study the functional groups of the components present in the DES and analyze the possible changes in the structure. The FTIR spectral data of pure urea were in accordance with the literature [82] showing the vibrational bands of the NH_2_ group at 3500–3200 cm^−1^, while the carbonyl stretching vibration is usually observed in the range of 1680–1650 cm^−1^. The vibrational bands at 1623 and 1461 cm^−1^ correspond to NH and C–N bending, respectively, and C–N stretching vibration is usually found in the range of 1300–1000 cm^−1^.

**Figure 5:**
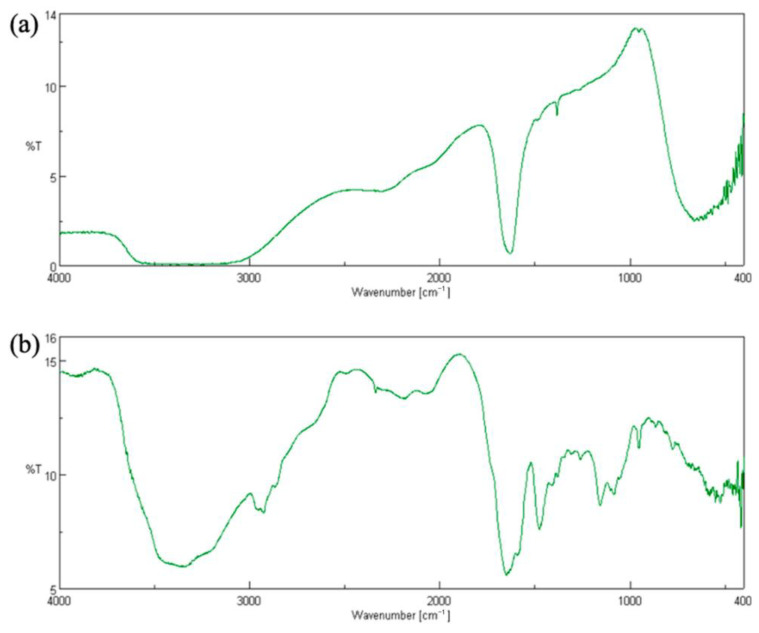
FT-IR spectra of (**a**) ErCl_3_·6H_2_O and (**b**) DES-4.

The FTIR spectrum of ErCl_3_·6H_2_O (Figure 5a) exhibited an absorption band around 3500–3100 cm^−1^ associated with the stretching vibrations of hydrogen atoms bound to oxygen in water molecules and around 1600 cm^−1^ (H–O–H bending vibration) indicating the presence of water molecules. The stretching vibrations of Er–Cl bonds appear in the lower wavenumber region, below 700 cm^−1^.

The coordination between erbium chloride hexahydrate and urea in DES-4 can indeed influence the stretching vibrations observed in the FTIR spectrum and are presented in Figure 5b.

In general, when erbium coordinates with urea or any ligand, it can lead to shifts or changes in the vibrational frequencies compared to the free ligand or metal salt. Coordination between erbium trichloride with urea resulted in shifts or splitting of the urea-related bands, such as the carbonyl (C=O) and amino (N–H) stretching vibrations, depending on the coordination mode. The FTIR spectrum for the DES-4 displays all bands corresponding to the functional groups of both constituents; however, a new peak below 2900 cm^−1^ appeared, which might suggest that new bonds are formed by coordination between urea and erbium trichloride. The characteristic vibrational modes in urea, including the carbonyl (C=O) stretching around 1650–1600 cm^−1^ and the N–H stretching around 3400–3200 cm^−1^, are influenced by the coordination with erbium ions and the presence of water molecules. The carbonyl vibration in urea showed a shift from 1678 cm^−1^ and overlaps with the bands in the FTIR of erbium trichloride, indicating that the coordination bonds were mainly formed between the oxygen of the carbonyl in urea and the metal ion.

### 3.3. Synthesis of Cellulose Carbamate

The role of DES-4 as a solvent, reagent, and catalyst was assessed in the preparation of cellulose carbamate. The presence of a large excess of urea in this DES-4 indicates that consumption of urea in the cellulose carbamate preparation does not disrupt the DES solvent system. Various experiments were conducted under different conditions to identify the most suitable procedure for obtaining carbamate, as schematically summarized in Table 2.

**Table 2:** Optimization data for the synthesis of CC.

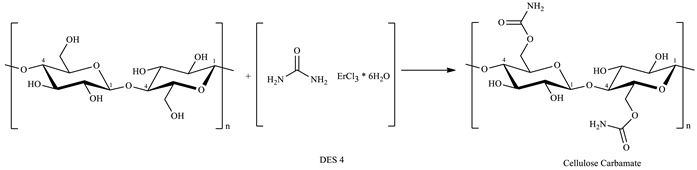					
Sample	Substrate	CC Preparation Method	Reaction Time (min)	N (%) ^a^	Degree of Substitution ^b^
1	Cellulose + H_2_O	Vacuum evaporation	60	1.59	0.19
2	Cellulose + H_2_O	US	30	1.42	0.17
3	Dried cellulose	US	30	0.86	0.10
4 ^c^	Cellulose + urea	Vacuum evaporation	60	0.27	0.05

^a^ value obtained by elemental analysis; ^b^ calculated from elemental analysis results; ^c^ experiment carried out in water and in the absence of ErCl_3_·6H_2_O.

In the first attempt, a suspension of microcrystalline cellulose (MCC) in water was added to DES-4. The mixture was stirred using a rotary evaporator under reduced pressure at 50 °C for 1 h, resulting in a pink homogeneous mixture. The obtained mixture was further dried in an oven at 100 °C for 24 h and then in contact with an oil bath at 150 °C for 1 h. The product, dry and adhering to the flask walls, was recovered by washing with a 12 M HCl solution, followed by filtration and additional washing with distilled water. The excess reagents and DES components were removed, and the filtered product was dried using a vacuum pump. A white material was obtained (Table 2, sample 1).

The FTIR spectra of dried cellulose and cellulose carbamate provide valuable insights into the chemical changes associated with the carbamation process. Dried cellulose typically exhibits characteristic peaks in the FTIR spectrum corresponding to the hydroxyl groups at around 3600–3200 cm^−1^ and the cellulose backbone vibrations [83,84]. Furthermore, C–O stretching around 1000–1300 cm^−1^ is predominant.

**Figure 6:**
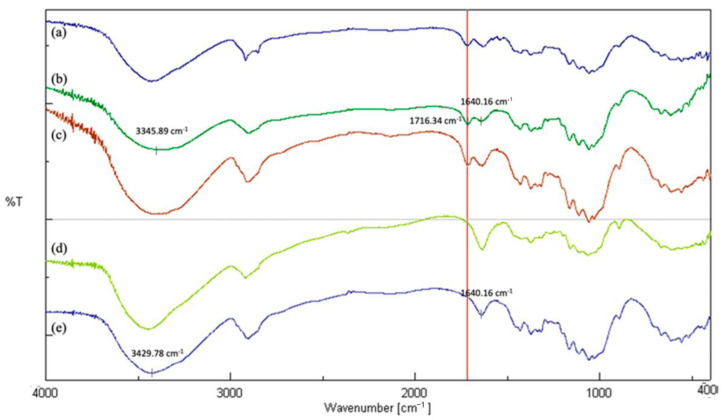
FT-IR spectra of (**a**) sample 2 (N% = 1.42, DS = 0.17), (**b**) sample 1 (N% = 1.59, DS = 0.19), (**c**) sample 3 (N% = 0.86, DS = 0.10), (**d**) sample 4 (N% = 0.27, DS = 0.05), and (**e**) starting dried cellulose.

Upon carbamation, a distinctive peak emerges in the carbonyl stretching region at 1716.34 cm^−1^, signaling the introduction of carbamate groups (Figure 6). This peak is attributed to the C=O stretching vibration of the carbonyl group in the carbamate functionality. The presence of this carbonyl peak in cellulose carbamate confirms the successful functionalization of cellulose with carbamate groups. The intensity and position of the carbonyl peak can provide information about the extent of functionalization and the nature of the carbamate groups introduced. This observation aligns with published literature data [77]. The absorption of cellulose hydroxyl groups at 3429.78 cm^−1^ decreases during carbamate formation, consistent with the formation of carbamates, especially with the primary OH groups [85].

In the second experiment, the synthesis of CC was attempted using ultrasonication conditions to enhance mixture homogeneity (Table 2, sample 2). A suspension of MCC in water was added to DES-4, and the reaction was assisted by ultrasound for 30 min, followed by stirring under reduced pressure using a rotary evaporator at 50 °C for 1 h. Subsequent purification treatments afforded a solid material that was analyzed by FTIR. The FT-IR spectrum of the modified cellulose revealed, in this case as well, the presence of the characteristic absorption peak due to the stretching vibration of the urethane carbonyl.

To propose an even more eco-sustainable and convenient synthetic procedure, the third experiment was carried out in the absence of water using ultrasonication (Table 2, sample 3). Although water is often suggested as an environmentally friendly additive to regulate DES viscosity and polarity, it can dramatically alter the DES structure due to the rupture of hydrogen bonds between the initial constituents [86]. Cellulose was directly suspended in the previously prepared DES-4, and the mixture was subjected to ultrasonication for 1 h. The final purification treatments afforded a white powder.

To verify the need for the catalyst, erbium trichloride, in the synthesis of cellulose carbamate, a control experiment was conducted in its absence, using only water as the reaction medium (Table 2, sample 4). Cellulose was suspended in water, and urea was subsequently added. The reaction was assisted by ultrasound for 1 h. The product obtained from subsequent purification treatments appeared as a copper-colored material. The FT-IR spectrum of the sample did not reveal the presence of the typical peak due to the urethane carbonyl, indicating that urea did not functionalize the primary hydroxyl of cellulose.

The carbonyl absorption in the FTIR spectra correlates with the nitrogen content, with a higher percentage of nitrogen resulting in a greater absorption peak. To verify this statement, the nitrogen content value (N%) of CC samples was determined using elemental analysis and the N% for each sample has been reported in detail in Table 2. These values were used to calculate the DS of hydroxyl groups on cellulose by carbamate groups and to confirm that the first experiment (sample 1) furnished the best experimental conditions for the preparation of CC with the highest nitrogen content (1.59%) that corresponded to a DS of 0.19.

## 4. Discussion

**Scheme 2:**
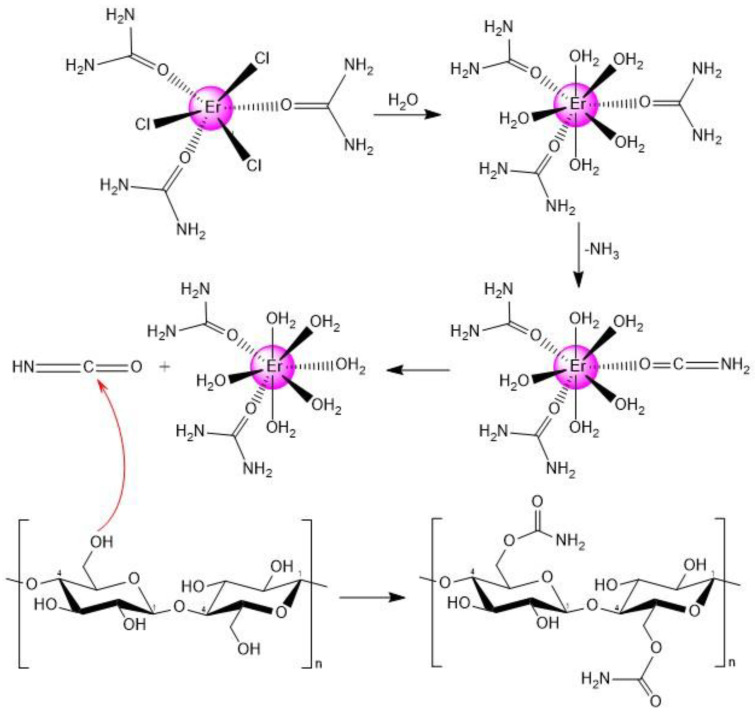
Hypothetical synthesis of cellulose carbamate using DES-4. In the scheme, for simplicity, the charges have been omitted.

Cellulose carbamate, synthesized using DES-4, was characterized by an FT-IR spectrometer. Analyzing the FT-IR spectra related to the different syntheses attempted, it can be concluded that well-defined spectra with a more evident peak due to the stretching of the carbonyl (C=O) of urethane were obtained from the experiments conducted in the presence of water. From these observations, we can hypothesize the formation of cellulose carbamate in a DES solvent according to the scheme described in Scheme 2.

From the reaction scheme, it can be observed how in the presence of water, the chloride ion is replaced in its bond with the erbium and the compound changes from hexacoordinate to an octacoordinated complex [81,82]. Erbium acting as a Lewis acid catalyst makes the amide carbon of urea more electrophilic. The formation of isocyanic acid is promoted by the development of an acid–base reaction that takes place between the amino group of urea and the water molecule coordinated with the erbium. The resulting ammonia causes the loss of a proton from the isocyanic cation with the consequent formation of ammonium chloride. Water favors the displacement of isocyanic acid from its coordination bond with erbium. Isocyanic acid at this point, being strongly reactive, in the free state allows the functionalization of cellulose. The presence of water, according to this hypothetical reaction scheme, is essential to allow the displacement first of the chloride ion and then of the isocyanic acid. Since the coordination bond between erbium and urea is shorter than the bonds with chlorine and the coordination bonds with isocyanic acid, it is evident that the action of water is exerted first in the replacement of chlorine atoms and subsequently in the replacement of the isocyanate formed, while it has no interference with coordination with urea being the strongest bond.

## 5. Conclusions

In conclusion, a novel type IV DES solvent was effectively prepared and used, exploiting its role as a solvent/catalyst and, at the same time, as an actual reactive component in the preparation of cellulose carbamate. Considering the comparatively lower toxicity of ErCl_3_·6H_2_O compared to common table salt, we advocate for the adoption of this innovative RDES composed of urea and erbium trichloride as an inexpensive, efficient, and environmentally compatible, non-toxic system for cellulose carbamate production. Notably, samples with a nitrogen content as high as 1.59% were achieved through this method. The potential for scalable implementation of this green synthesis approach in the future holds promise to produce environmentally friendly products, improving cellulose solubility and streamlining its derivatization process.

## Figures and Tables

**Table 1:** Preparation of DESs using ErCl_3_ (anhydrous) or ErCl_3_·6H_2_O (hexahydrate) and urea.

Entry	DES	Components	Molar Ratio	Preparation Method	Time (min)	Aspect
** 1 **	-	ErCl_3_ anhydrous: urea	1:1	Vacuum evaporation	60	Pink solid
** 2 **	-	ErCl_3_ anhydrous: urea	1:3	Vacuum evaporation	60	Pink solid
** 3 **	-	ErCl_3_·6H_2_O: urea	1:3	Vacuum evaporation	60	Pink highly viscous liquid with particles in suspension
** 4 **	DES-1	ErCl_3_·6H_2_O: urea (+ 20% water)	3:10	Vacuum evaporation	60	Clear pink viscous liquid
** 5 **	-	ErCl_3_·6H_2_O: urea (+20% water)	3:10	Ultrasonication-assisted preparation	30	Clear pink viscous liquid with particles in suspension
** 6 **	DES-2	ErCl_3_·6H_2_O: urea (+20% water)	3:10	Ultrasonication-assisted preparation	60	Clear pink viscous liquid
** 7 **	DES-3	ErCl_3_·6H_2_O: urea	3:10	Heating and stirring	60	Clear pink viscous liquid
** 8 **	DES-4	ErCl_3_·6H_2_O: urea	3:10	Ultrasonication-assisted preparation and vacuum evaporation	120	Clear pink viscous liquid

## Data Availability

Data are contained within the article.
